# A Minimally Invasive Approach to Caecal Volvulus: A Rare Complication of Colonoscopy

**DOI:** 10.7759/cureus.96046

**Published:** 2025-11-03

**Authors:** David Heath, Kyungchul Kim, David J Solomon, Abraham Jacob

**Affiliations:** 1 General Surgery, Bunbury Regional Hospital, Perth, AUS; 2 General Surgery, Rockingham General Hospital, Perth, AUS; 3 Orthopedics, Royal Victoria Hospital, Belfast, IRL; 4 General and Colorectal Surgery, Royal Perth Hospital, Perth, AUS

**Keywords:** bowel obstruction, caecal volvulus, colonoscopy complications, laparoscopic surgery, minimally invasive surgery, postoperative complications, right hemicolectomy, surgical emergency

## Abstract

Caecal volvulus following colonoscopy represents an extremely rare complication with only a few cases documented in the literature. We report the case of a 67-year-old woman who developed caecal volvulus 24 hours following routine colonoscopy with polypectomy. The patient presented with progressive cramping abdominal pain and nausea. Computed tomography revealed caecal displacement to the right upper abdomen without proximal ascending colon distension. Laparoscopic exploration confirmed caecal volvulus with ischaemia but no perforation. A laparoscopic right hemicolectomy was successfully performed, representing the first reported case managed with this minimally invasive approach. The patient recovered without complications and was discharged on postoperative day 5. This case emphasises the importance of maintaining high clinical suspicion for caecal volvulus in patients presenting with abdominal pain following colonoscopy. Early recognition and prompt laparoscopic intervention can prevent progression to bowel necrosis and perforation while offering superior outcomes compared to traditional open surgical approaches. The minimally invasive technique provides excellent visualisation, reduced morbidity, and faster recovery times, establishing it as the preferred management approach when surgical expertise is available.

## Introduction

Caecal volvulus is a rare but potentially life-threatening condition characterised by torsion or twisting of the caecum and ascending colon about their mesenteric axis, leading to bowel obstruction and potential vascular compromise [1]. The condition has an incidence of 2.8-7.1 per million people per year in the general population, representing approximately 1-2% of all large bowel obstructions and 25-40% of all colonic volvuli [2,3]. However, caecal volvulus as a complication of colonoscopy is extraordinarily rare, with only four cases documented in the literature prior to 2015 and fewer than 10 cases reported to date [4].

Despite the rarity of this complication, existing literature reveals significant limitations in current management approaches. Historical reports from the 1980s and 1990s documented poor outcomes with high morbidity and mortality rates, primarily attributed to delayed diagnosis and the exclusive use of open surgical techniques [5,6]. These early cases were characterised by prolonged hospital stays, increased complication rates, and suboptimal functional recovery. Furthermore, the existing literature lacks a comprehensive evaluation of minimally invasive surgical approaches for managing this specific complication, with all previously reported cases managed through traditional open surgical methods.

This research gap is particularly significant given the documented advantages of laparoscopic techniques in emergency colorectal surgery, including superior visualisation, reduced surgical trauma, and improved patient outcomes [7,8]. However, no previous study has evaluated the feasibility, safety, or outcomes of laparoscopic management specifically for post-colonoscopy caecal volvulus, representing a clear knowledge deficit in the current literature.

This case report aims to address these limitations by (1) documenting the first successful laparoscopic management of post-colonoscopy caecal volvulus, (2) demonstrating the technical feasibility and safety of minimally invasive surgical intervention in this emergency setting, (3) providing evidence for improved patient outcomes compared to historical open surgical approaches, and (4) establishing a new standard of care for this rare but serious complication.

This paper makes several specific contributions to the surgical literature. First, it establishes laparoscopic right hemicolectomy as a viable and superior treatment option for post-colonoscopy caecal volvulus, differentiating it from existing literature that exclusively reports open surgical management. Second, it provides detailed technical insights into the laparoscopic approach for emergency colorectal surgery in the setting of acute volvulus, offering practical guidance for surgeons managing similar cases. Third, it demonstrates significantly improved outcomes compared to historical reports, with reduced hospital stay, absence of complications, and complete functional recovery. Finally, it contributes to the broader understanding of minimally invasive emergency surgery by expanding the indications for laparoscopic intervention to include rare complications of routine procedures.

The pathophysiology of caecal volvulus involves abnormal mobility of the caecum and ascending colon, typically due to incomplete embryological fixation of the right colon to the posterior abdominal wall or acquired factors that increase colonic mobility [9]. During colonoscopy, several mechanisms may predispose to volvulus formation, including excessive air insufflation leading to colonic distension, vigorous manipulation during scope advancement, and mechanical trauma to the bowel wall and mesentery [10]. The combination of these factors in patients with underlying anatomical predisposition can result in the delayed presentation of caecal volvulus hours to days following the procedure.

Clinical presentation of caecal volvulus typically includes severe abdominal pain, often beginning in the lower abdomen and progressing to the right upper quadrant, accompanied by nausea, vomiting, and signs of bowel obstruction [11]. The pain is characteristically cramping or colicky in nature and progressively worsens as bowel ischaemia develops. Physical examination may reveal abdominal distension, localised tenderness, and hypoactive bowel sounds, though these findings can be subtle in early presentations.

Radiological diagnosis relies primarily on computed tomography (CT) imaging, which demonstrates characteristic findings including the "coffee bean" sign, "bird beak" sign, and "whirl sign" representing the twisted mesenteric vessels [12,13]. Plain abdominal radiographs may show caecal distension in the right lower quadrant with the classic "coffee bean" appearance, though CT remains the gold standard for the definitive diagnosis and assessment of bowel viability.

Traditional management of caecal volvulus has involved open surgical intervention, with options including detorsion with caecopexy, caecostomy, or right hemicolectomy depending on bowel viability and patient factors [14]. The development of advanced laparoscopic techniques has created potential opportunities for minimally invasive management, though this approach has not been previously reported for post-colonoscopy caecal volvulus.

## Case presentation

A 67-year-old woman presented to the emergency department with a 24-hour history of progressive cramping abdominal pain and associated nausea. The patient had undergone a routine screening colonoscopy with an uncomplicated polypectomy the previous day. Her medical history was unremarkable, with no previous abdominal surgeries or known gastrointestinal disorders. The colonoscopy had been performed under conscious sedation and was notable for diverticulosis of the sigmoid, descending, transverse, and ascending colon, with the successful removal of several small polyps.

The patient reported that symptoms began approximately 12 hours post-procedure, initially presenting as mild lower abdominal discomfort that progressively intensified and migrated to the right upper quadrant. The pain was described as severe, cramping in nature, and associated with nausea but no vomiting. She reported inability to pass flatus or have bowel movements since the onset of symptoms, raising concern for bowel obstruction.

On physical examination, the patient appeared uncomfortable but haemodynamically stable with normal vital signs. Abdominal examination revealed distension with focal tenderness in the lower abdomen and right upper quadrant. Peritonitic signs were present with guarding and rebound tenderness, particularly in the right iliac fossa. Bowel sounds were diminished but audible. Digital rectal examination revealed an empty rectum with no masses palpable.

Initial laboratory investigations showed a mild leucocytosis with a white blood cell count of 12.8×10⁹/L (normal range 4.0-11.0×10⁹/L), but other parameters, including haemoglobin, electrolytes, and liver function tests, were within normal limits. Lactate levels remained normal at 1.2 mmol/L, suggesting the absence of significant bowel ischaemia at presentation.

**Figure 1:**
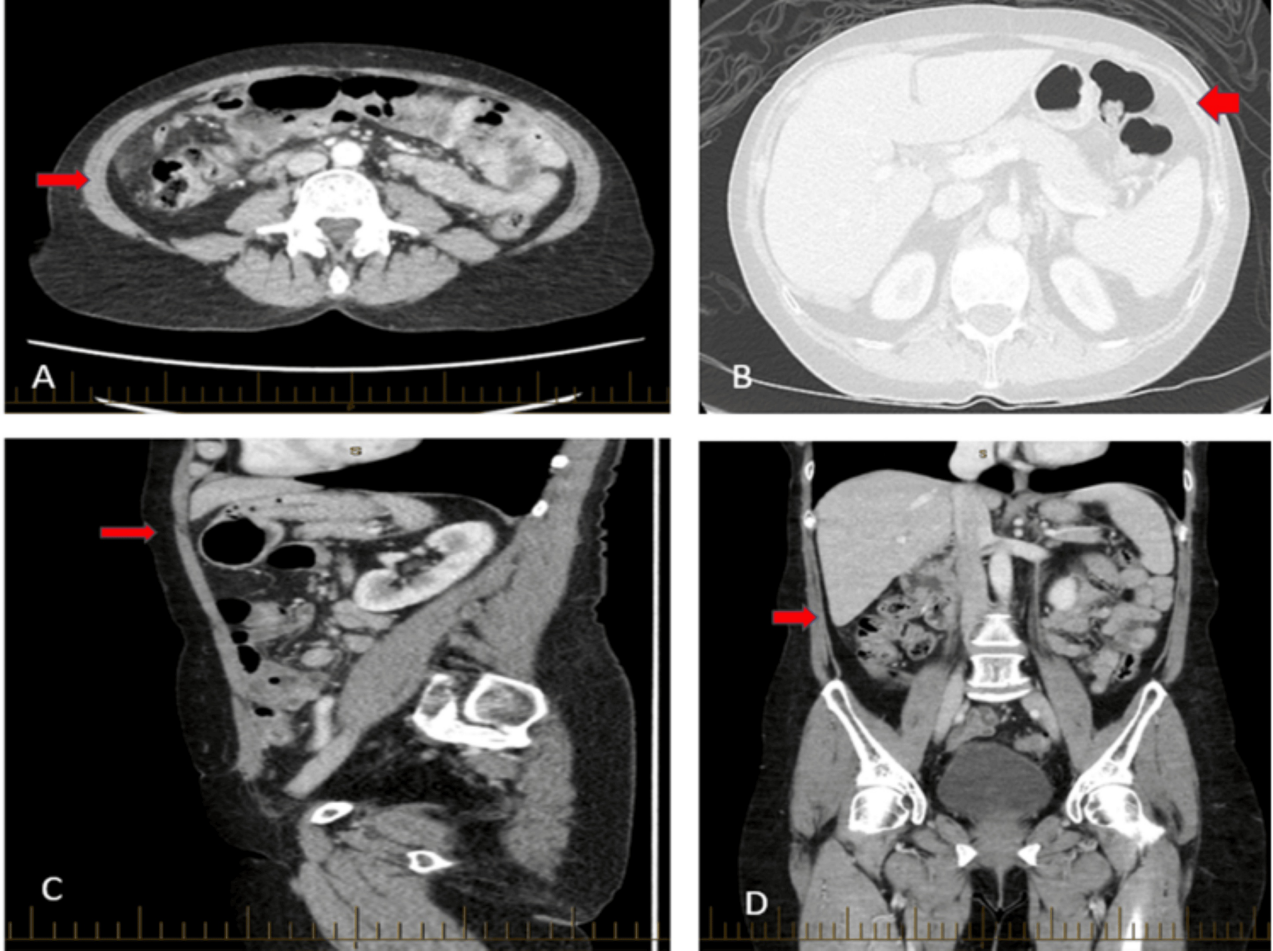
Computed tomography showing the caecum is displaced into the right upper abdomen, without distension of the proximal ascending colon

CT of the abdomen and pelvis with intravenous contrast demonstrated the caecum displaced into the right upper abdomen with characteristic features of volvulus. The imaging showed no distension of the proximal ascending colon, moderate fat stranding around the caecum, and the classic "whirl sign" indicating mesenteric torsion. Importantly, there was no evidence of bowel perforation or free intraperitoneal air (Figure 1). The CT findings, combined with the clinical presentation and recent colonoscopy history, established the diagnosis of caecal volvulus.

**Figure 2:**
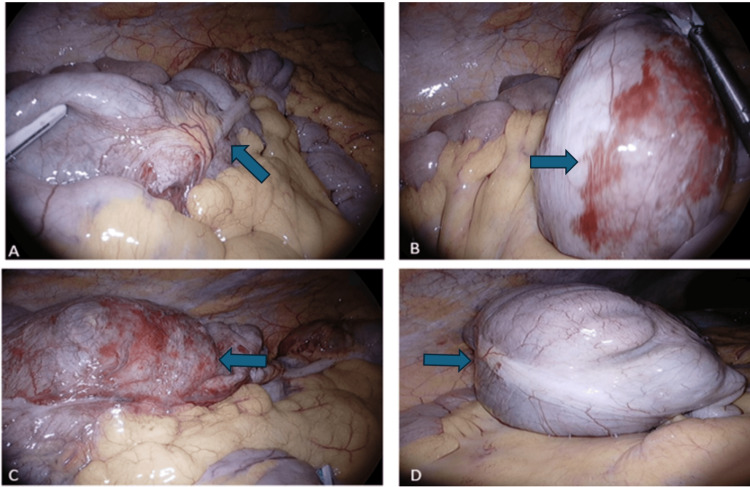
Intraoperative photos: (A) Laparoscopic view showing the twisted pedicle at the base of the caecal volvulus. (B) The dilated caecum demonstrating ischaemic changes with serosal haemorrhage. (C) Caecal volvulus with ulceration and surrounding ischaemia without evidence of perforation. (D) Appearance of the caecum after successful detorsion

Given the clinical and radiological findings, the patient was taken urgently to the operating theatre for laparoscopic exploration. Intraoperative findings confirmed caecal volvulus with the caecum twisted approximately 180 degrees about its mesenteric axis (Figure 2). The caecum appeared ischaemic with areas of serosal discolouration and surrounding inflammation, but remained intact without evidence of perforation. The bowel was successfully untwisted, revealing viable but compromised tissue that warranted resection to prevent future complications.

A laparoscopic right hemicolectomy was performed using a five-port technique with CO₂ insufflation. The procedure involved mobilisation of the right colon, division of the ileocolic and right colic vessels, and creation of a side-to-side ileocolic anastomosis using a linear stapling device. The specimen was extracted through a small Pfannenstiel incision, minimising abdominal wall trauma. The total operative time was 180 minutes with minimal blood loss.

Histopathological examination of the resected specimen confirmed acute ischaemic changes consistent with mechanical obstruction, with areas of mucosal ulceration and submucosal oedema. No evidence of malignancy was identified, and the surgical margins were clear. The postoperative course was uncomplicated, with the patient tolerating diet on postoperative day 2 and achieving normal bowel function by day 4. She was discharged home on postoperative day 5 in excellent condition. At the three-month follow-up, the patient remained asymptomatic with complete functional recovery and no evidence of complications.

## Discussion

This case represents the first reported successful management of post-colonoscopy caecal volvulus using a completely laparoscopic approach. The rarity of this complication, with fewer than 10 cases documented in the literature, underscores the importance of maintaining high clinical suspicion and prompt recognition in patients presenting with abdominal pain following colonoscopy [7,15-19].

The pathogenesis of caecal volvulus following colonoscopy involves multiple contributing factors that distinguish it from spontaneous cases. During colonoscopy, excessive air insufflation can lead to significant colonic distension, altering normal anatomical relationships and increasing intraluminal pressures [20]. The mechanical manipulation required for scope advancement, particularly in patients with redundant or mobile colonic segments, can predispose to subsequent torsion. Additionally, the sedation used during the procedure may mask early symptoms, leading to delayed recognition and presentation [21].

Historical cases of post-colonoscopy caecal volvulus, as demonstrated in Table 1, were associated with significant morbidity due to delayed diagnosis and the exclusive use of open surgical techniques. These early reports documented prolonged hospital stays (10-16 days), with some cases developing gangrenous or necrotic bowel requiring extensive resection [7]. The outcomes in these cases highlight the importance of early recognition and intervention to prevent progression to irreversible bowel compromise.

**Table 1:** Previously reported cases of caecal volvulus following colonoscopy CT: computed tomography

Author	Patient age/sex	Symptom onset following colonoscopy	Method of diagnosis	Treatment of caecal volvulus	Clinical outcome
Anderson et al. [7]	71/F	48 hours	Intraoperative	Laparotomy with subtotal colectomy	Fistula formation; discharged 55 days postoperatively
Radin and Halls [15]	55/F	24 hours	Abdominal X-ray and barium enema	Laparotomy	Death from sepsis due to faecal peritonitis
Amidon and Story [16]	62/M	24 hours	Abdominal X-ray	Laparotomy with caecectomy with primary anastomosis	Uncomplicated post-op recovery
Viney et al. [17]	58/F	12 hours	Intraoperative	Laparotomy with right hemicolectomy and primary anastomosis	Uncomplicated post-op recovery
Beltzer et al. [18]	52/M	4 hours	CT of the abdomen	Laparotomy with caecopexy	Failure of caecopexy with laparotomy and caecal resection; discharged day 5 post-second procedure
López et al. [19]	56/F	12 hours	CT of the abdomen	Exploratory laparotomy and right hemicolectomy	Uncomplicated post-op recovery

The laparoscopic approach employed in this case provided several documented advantages based on established evidence from emergency colorectal surgery literature. Studies have demonstrated that laparoscopic techniques in emergency settings result in reduced surgical site infections, decreased postoperative pain, shorter hospital stays, and faster return to normal activities compared to open approaches [22]. In this specific case, these benefits were evident through the patient's rapid recovery, absence of complications, and discharge on postoperative day 5, representing a significant improvement compared to historical cases.

From a technical perspective, laparoscopic right hemicolectomy for caecal volvulus presents unique challenges that require advanced surgical expertise. The distended and inflamed bowel can complicate port placement and initial insufflation, necessitating careful trocar insertion and gradual CO₂ introduction to avoid inadvertent bowel injury. The inflammatory changes and tissue oedema associated with volvulus can obscure normal anatomical planes, requiring meticulous dissection and identification of critical structures, including the ureter, duodenum, and major vascular pedicles.

The operative technique employed in this case utilised a standardised five-port approach with strategic port placement to optimise visualisation and instrument triangulation. The medial-to-lateral mobilisation technique was particularly advantageous, allowing early identification and control of the ileocolic vessels before extensive mobilisation of inflamed tissues. This approach minimises the risk of inadvertent injury to adjacent structures and reduces operative bleeding, which can be particularly problematic in the setting of acute inflammation.

The creation of the ileocolic anastomosis using intracorporeal techniques has been shown in multiple studies to provide excellent luminal diameter matching between the ileum and colon while minimising tension on the anastomotic line [23]. The laparoscopic approach allows for the precise placement of the enterotomies and optimal visualisation during stapler insertion, potentially reducing the risk of anastomotic complications compared to extracorporeal techniques that may compromise visualisation in challenging cases.

Evidence from systematic reviews and meta-analyses supports the benefits of laparoscopic approaches in emergency colorectal surgery. A recent meta-analysis by Veldkamp et al. demonstrated significantly reduced morbidity, shorter hospital stays, and improved quality of life scores in patients undergoing emergency laparoscopic colorectal procedures compared to open surgery [24]. These findings support the application of minimally invasive techniques in emergency settings when appropriate expertise is available.

The economic implications of minimally invasive management have been well-documented in health economic studies. Shorter hospital stays, reduced complication rates, and faster return to normal activities result in significant cost savings for healthcare systems and improved quality of life for patients [24]. In this case, the patient's discharge on postoperative day 5 compares favourably to historical reports of open surgery for similar conditions, where hospital stays ranged from 10 to 16 days.

The learning curve associated with emergency laparoscopic surgery requires consideration in the broader context of surgical training and service delivery. The successful management of complex emergency conditions laparoscopically demands not only technical proficiency but also sound surgical judgment in patient selection and timing of intervention. The availability of experienced laparoscopic surgeons and appropriate equipment in emergency settings remains a limiting factor in many institutions, highlighting the need for continued investment in surgical training and infrastructure development.

Future directions in the management of caecal volvulus may include the development of enhanced imaging techniques for earlier diagnosis, refinement of laparoscopic surgical techniques, and investigation of prophylactic measures in high-risk patients undergoing colonoscopy. The role of artificial intelligence in pattern recognition from CT imaging may facilitate more rapid diagnosis, while advances in surgical robotics could further enhance the precision and accessibility of minimally invasive techniques.

The implications for colonoscopy practice include heightened awareness of this rare but serious complication, particularly in patients with known risk factors such as previous abdominal surgery, chronic constipation, or anatomical variants. While the absolute risk remains extremely low, the potential for serious morbidity necessitates careful patient counselling and prompt recognition of concerning symptoms in the post-procedure period.

Regarding limitations as a single case report, the findings cannot be generalised to broader patient populations. The absence of a control group prevents direct comparison with traditional open surgical approaches. Additionally, the three-month follow-up period is insufficient for evaluating long-term outcomes. Future multicentre studies would be necessary to validate this minimally invasive approach for this rare condition.

## Conclusions

Caecal volvulus following colonoscopy represents an extremely rare but potentially serious complication requiring urgent surgical intervention, with this case demonstrating the successful application of laparoscopic right hemicolectomy as a safe and effective treatment option. The laparoscopic approach provided superior visualisation and precise surgical technique while minimising trauma, with early recognition and timely intervention preventing progression to bowel necrosis and optimising patient recovery compared to traditional open surgical management. Clinicians should maintain awareness of this rare complication and ensure appropriate post-procedure patient counselling. The availability of advanced laparoscopic surgical expertise in emergency settings represents a significant advancement in managing complex surgical emergencies, establishing laparoscopic right hemicolectomy as the preferred approach for this condition when technical resources are available.

## References

[1] Hasbahceci M, Basak F, Alimoglu O (2012). Cecal volvulus. Indian J Surg.

[2] Koutras G, Pandey N, Sawhney H, Mehak S, Hsieh J (2023). An unexpected twist: a case of cecal volvulus following colonoscopy. Am J Gastroenterol.

[3] Solis Rojas C, Vidrio Duarte R, García Vivanco DM, Montalvo-Javé EE (2020). Cecal volvulus: a rare cause of intestinal obstruction. Case Rep Gastroenterol.

[4] Shah N, Patel H, Patel V, Spira R (2016). Cecal bascule after colonoscopy - case report and review of literature. N Am J Med Sci.

[5] Sharma C, Shekhar S, Kumar S, Chaudhary R (2012). Gangrenous cecal volvulus complicating puerperium: is the delay in diagnosis really inevitable?. Case Rep Obstet Gynecol.

[6] Sedik A, Bar E, Ismail M (2015). Cecal volvulus: case report and review of literature. Saudi Surg J.

[7] Anderson JR, Spence RA, Wilson BG, Hanna WA (1983). Gangrenous caecal volvulus after colonoscopy. Br Med J.

[8] Mason RJ, Moazzez A, Moroney JR, Katkhouda N (2012). Laparoscopic vs open appendectomy in obese patients: outcomes using the American College of Surgeons National Surgical Quality Improvement Program database. J Am Coll Surg.

[9] Delabrousse E, Sarliève P, Sailley N, Aubry S, Kastler BA (2007). Cecal volvulus: CT findings and correlation with pathophysiology. Emerg Radiol.

[10] Tsushimi T, Kurazumi H, Takemoto Y (2008). Laparoscopic cecopexy for mobile cecum syndrome manifesting as cecal volvulus: report of a case. Surg Today.

[11] Mahruqi GA, Ebrahim MA, Aghbari SA, Kindi SA, Koliyada SV (2019). Cecal volvulus: a case report and literature review. Int J Innov Res Med Sci.

[12] Vandendries C, Jullès MC, Boulay-Coletta I, Loriau J, Zins M (2010). Diagnosis of colonic volvulus: findings on multidetector CT with three-dimensional reconstructions. Br J Radiol.

[13] Rosenblat JM, Rozenblit AM, Wolf EL, DuBrow RA Findings of cecal volvulus at CT.

[14] Katoh T, Shigemori T, Fukaya R, Suzuki H (2009). Cecal volvulus: report of a case and review of Japanese literature. World J Gastroenterol.

[15] Radin DR, Halls JM (1986). Cecal volvulus: a complication of colonoscopy. Gastrointest Radiol.

[16] Amidon PB, Story RK Jr (1993). Cecal volvulus after colonoscopy. Gastrointest Endosc.

[17] Viney R, Fordan SV, Fisher WE, Ergun G (2002). Cecal volvulus after colonoscopy. Am J Gastroenterol.

[18] Beltzer C, Geiger A, Schmidt R, Danz B, Maier A, Karpa R, Dikopoulos N (2017). A rare case of coecal volvulus after colonoskopy due to a mobile coekum - diagnosis, surgical therapy and postoperative complications [Article in German]. Z Gastroenterol.

[19] López JE, Echevarria YL, Mursuli AL, López Rodríguez PR (2024). Complicated sigmoid colon volvulus. Case presentation. Open Access J Surg.

[20] Schwenk W, Haase O, Neudecker J, Müller JM (2005). Short term benefits for laparoscopic colorectal resection. Cochrane Database Syst Rev.

[21] Milsom JW, Böhm B, Hammerhofer KA (1998). A prospective, randomized trial comparing laparoscopic versus conventional techniques in colorectal cancer surgery: a preliminary report. J Am Coll Surg.

[22] Ballantyne GH, Brandner MD, Beart RW, Ilstrup DM (1985). Volvulus of the colon: incidence and mortality. Ann Surg.

[23] Zabeirou AA, Belghali H, Souiki T (2019). Acute cecal volvulus: a diagnostic and therapeutic challenge in emergency: a case report. Ann Med Surg.

[24] Veldkamp R, Kuhry E, Hop WC (2005). Laparoscopic surgery versus open surgery for colon cancer: short-term outcomes of a randomised trial. Lancet Oncol.

